# 

**Published:** 1882-06

**Authors:** 


					﻿WjloijE Number,
CCCCXLIX.
June, 1882.
Number 6,
Vol. XLIV.
THE
CHICAGO
MEDICAL
J ournal and Examiner
EDITORS:
N. S. DAVIS, M.D., LL.D. JAS. NEVINS HYDE, A.M., M.D.
DANIEL R. BROWER, A.M., M.D.
CHICAGO:
PUBLISHED BY THE CHICAGO MEDICAL PRESS ASSOCIATION,
188 Clark Street.
^*Read the Notice of this Journal among Advertisements.
				

